# A Review of the Literature on Videoscopic and Robotic Inguinal–Iliac–Obturator Lymphadenectomy in Patients with Cutaneous Melanoma

**DOI:** 10.3390/jcm13237305

**Published:** 2024-12-01

**Authors:** Matteo Matteucci, Paolo Bruzzone, Sabrina Pinto, Piero Covarelli, Carlo Boselli, Georgi I. Popivanov, Roberto Cirocchi

**Affiliations:** 1 Department of General Surgery, University of Milan, 20122 Milan, Italy; matteo.matteucci@unimi.it (M.M.); sabrina.pinto@unimi.it (S.P.); 2 Department of General and Specialist Surgery, University of Roma La Sapienza, 00185 Rome, Italy; paolo.bruzzone@uniroma1.it (P.B.); carlo.boselli@unipg.it (C.B.); 3 Department of General Surgery, University of Perugia, 06129 Perugia, Italy; piero.covarelli@unipg.it; 4 Department of Surgery, Medical Military Academy of Sofia, 1606 Sofia, Bulgaria

**Keywords:** melanoma, cutaneous melanoma, robotic surgery, robotic inguinal, iliac, and obturator lymphadenectomy, minimally invasive surgery

## Abstract

Inguinal–iliac–obturator lymph node dissection is essential in the treatment of patients with cutaneous melanoma exhibiting the clinical or radiological involvement of pelvic lymph nodes. The open procedure is associated with elevated mortality rates. Numerous minimally invasive approaches have been suggested to mitigate the impact of this surgery on the patient’s quality of life. The preliminary findings of robotic-assisted dissection have been documented in the literature. They demonstrate a decrease in potential issues linked to robotic-assisted treatments as compared to open or video laparoscopic methods. No implications have been reported for long-term oncological outcomes. The present study compares the outcomes in 64 patients with robotic procedures, 187 with videoscopic procedures, and 83 with open pelvic lymph node dissection (PLND). However, the quality of evidence is too low to draw any valid conclusions. The available literature shows that a robotic procedure is feasible and has similar complication rates and oncological outcomes to other methods. The reason for the shorter operative time is not clear, but is associated with lower hospital costs. It is probable that, from a surgeon’s point of view, robotic techniques offer several advantages over videoendoscopic techniques, such as three-dimensional imaging, ergonomic control, and tools that mimic human hand movements. Randomized controlled trials are necessary to validate the benefits of robotic inguinal–iliac–obturator lymph node dissection (RIIOL) compared to videoscopic and open procedures, but the recruitment rate is very low because of the restricted indications for lymph node dissection against the background of the continuously evolving system of therapy.

## 1. Introduction

Cutaneous melanoma represents, in Italy, the second most frequent cancer in the male sex and the third most frequent cancer in the female sex. The risk of developing melanoma is 1.5% in males and 1.2% in females [1].

In the standard treatment for cutaneous melanoma, local wide excision and sentinel lymph node biopsy are used for patients with cutaneous melanoma pT1b or higher. For many years, complete lymph node dissection has been the standard approach for patients with metastatic sentinel lymph nodes.

According to AIOM guidelines [1] and to NCCN guidelines [2] on cutaneous melanoma, complete lymph node dissection is the treatment of choice in patients with clinically and/or radiologically involved lymph nodes.

According to the NCCN guidelines of 2024 [2], lymph node dissection is also indicated in clinically positive LNs after neoadjuvant immunotherapy, targeted therapy, or both.

**Figure 1:**
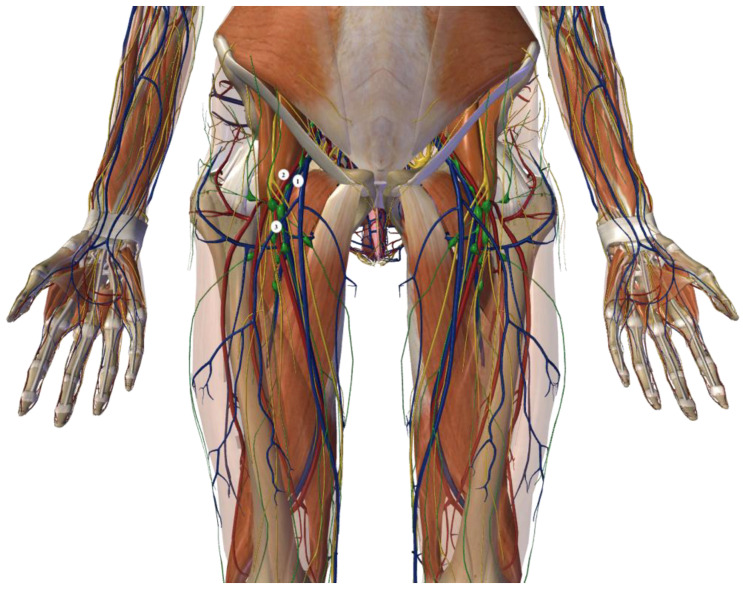
The location of lymph nodes during the inguinal step of the IIOL procedure. 1: femoral vein; 2: femoral artery; 3: inguinal lymph nodes. The figure was created with the Zigote Body program.

**Figure 2:**
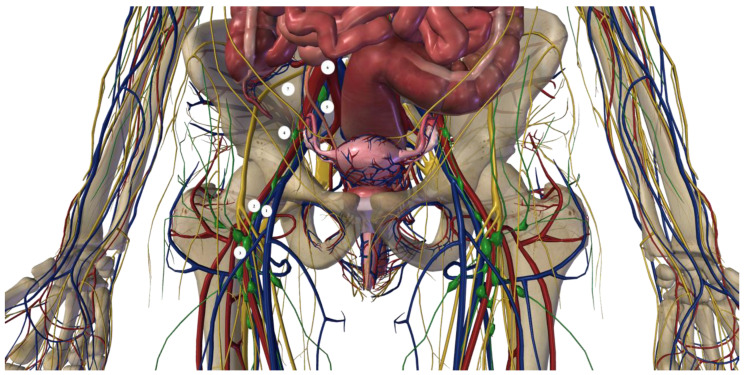
The location of lymph nodes during the inguinal step and intra-abdominal step of the IIOL procedure. 1: femoral vein; 2: femoral artery; 3: inguinal lymph nodes; 4: iliac lymph nodes; 5: obturator lymph nodes; 6: bifurcation of iliac artery; 7: obturator nerve. The figure was created with the Zigote Body program.

Inguinal–iliac–obturator lymphadenectomy (IIOL) involves the removal of the lymph nodes and lymphatic tissue associated with the course of the external femoral and iliac vessels. The upper limit of the procedure is represented by the bifurcation of the common iliac artery into the external and internal iliac arteries. Instead, the lower limit is represented by the caudal apex of the Scarpa’ triangle. Therefore, the dissection concerns two different anatomical districts: the iliac region, which is an intra-abdominal district, and the extra-abdominal inguinal–obturator region (Figure 1 and Figure 2).

This surgery is associated with elevated morbidity rates, often resulting in seroma or lymphocele formation, wound complications, and persistent lymphoedema, significantly diminishing patients’ quality of life. The incidence of severe complications ranges from 50% to 90% in cases of open dissection [3]. To mitigate these difficulties, novel minimally invasive procedures have been developed. One of these methods is robotic inguinal–iliac–obturator lymphadenectomy (RIIOL).

### 1.1. Open Inguinal–Iliac–Obturator Lymph Node Dissection Technique

This surgery is performed under general anesthesia. Before the operation, a bladder catheter is placed: this procedure empties the bladder and reduces iatrogenic damage.

The patient is placed in a supine position, with the limb to be operated on moderately bent to the knee and rotated externally on the hip joint. This position facilitates the exposure of the femoral triangle.

A lozenge incision is executed and is aligned along an oblique trajectory from moving superior to inferior and latero-medially. It originates 5 cm superiorly and medially at the iliac spine and extends inferiorly to the apex of the Scarpa triangle, approximately 10–12 cm from the inguinal ligament. The skin flaps are established. In the inguinal phase of the process, the medial boundary is defined by the pubic tubercle and the long adductor muscle, while the lateral boundary is marked by the anterosuperior iliac spine and the sartorius muscle. Consequential to the incision of the lata fascia, the lymph node tissue is meticulously dissected until reaching the femoral vein, from which the adventitia is separated. The saphenous–femoral junction is identified and dissected. The femoral artery is isolated, followed by the removal of the initial surgical specimen. The subsequent step of the treatment involves the iliac–obturator interval, commencing with the incision of the inguinal ligament. The anatomical and surgical boundaries of this phase are delineated by the bifurcation of the iliac arteries superiorly, the obturator nerve inferiorly, the external iliac artery laterally, and the inguinal ligament medially. 

### 1.2. Videoscopic Inguinal–Iliac–Obturator Lymph Node Dissection Technique

**Figure 3:**
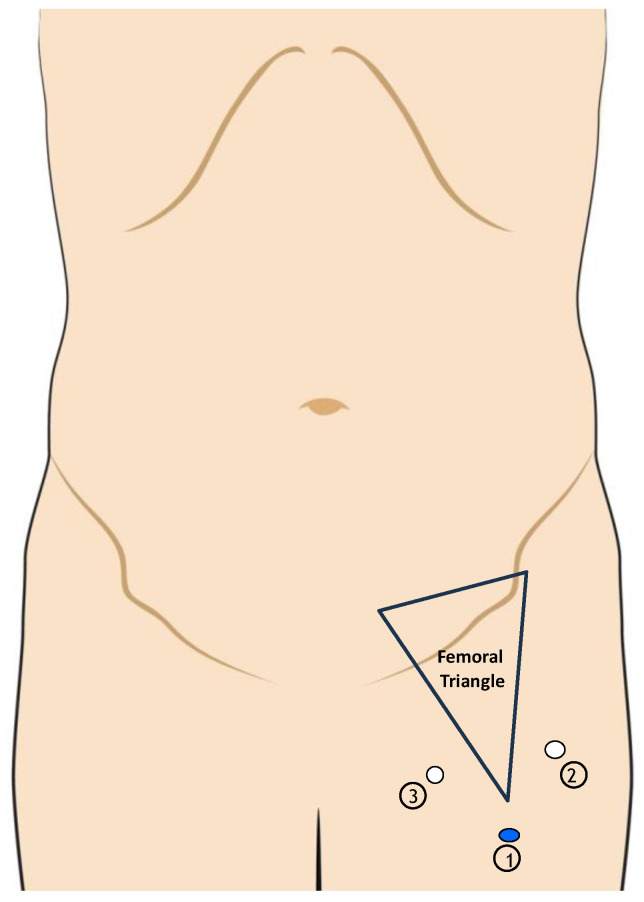
Port placement during the pelvic step of videoscopic inguinal–iliac–obturator dissection. 1: the first trocar located 2 cm above the triangle of Scarpa; 2: the second port located 2 cm medially to the abductors; 3: the third port 2 cm laterally to the sartorius muscle.

The inguinal step is performed with three ports: the first one is located 2 cm above the apex of Scarpa triangle. The other two ports are located, respectively, 2 cm medial to the abductors and 2 cm lateral to the sartorius muscle. The operative field is established via blunt dissection under the superficial fascia while maintaining a carbon dioxide pressure of 12 mmHg throughout the process. This is performed using an ultrasonic dissector (Figure 3).

**Figure 4:**
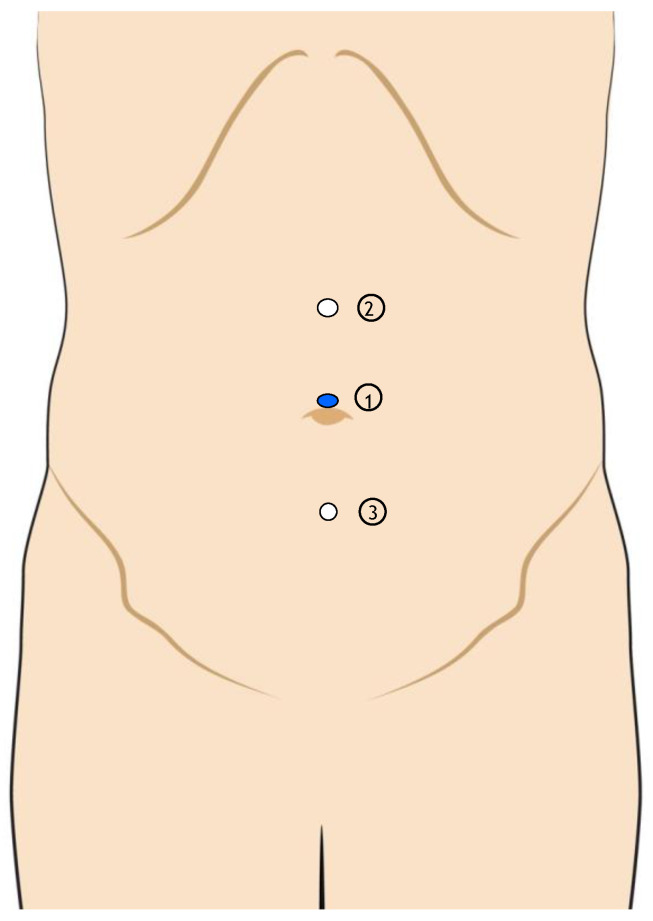
Port placement during the iliac–obturator step of videoscopic inguinal–iliac–obturator dissection. 1: Hasson trocar in the periumbilical area; 2–3: the others ports.

The iliac–obturator procedure involves the placement of three distinct trocars: a Hasson trocar in the periumbilical area and two supplementary trocars (5 mm and 10 mm), positioned laterally to the abdominal midline. A pneumoperitoneum of 12 mmHg is sustained (Figure 4).

### 1.3. Robotic Inguinal–Iliac–Obturator Lymph Node Dissection Technique

**Figure 5:**
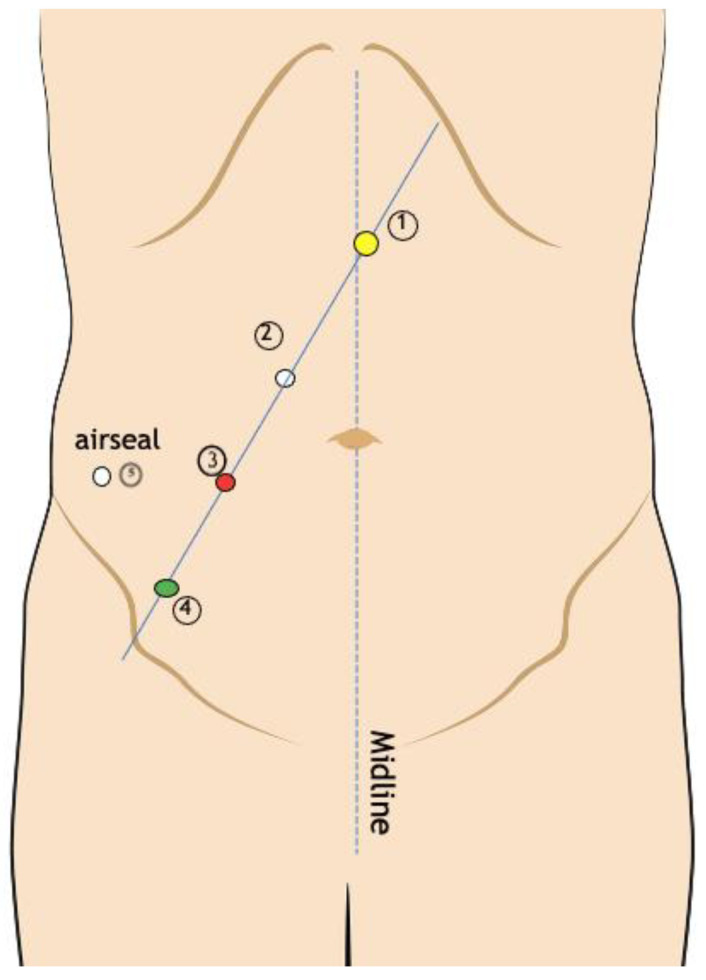
Port placement during the pelvic step of robotic inguinal and iliac–obturator dissection. 1: 8 mm periumbilical port (the first port that is inserted). 2–3–4: 8 mm ports, placed under laparoscopic vision, spaced 8 cm from each other. 5: 12 mm service port.

The patient is positioned supine, and the operating table is inclined 30° in the Trendelenburg position. Three preoperative injections of 1 mL of indocyanine green (ICG) are recommended for the assessment of lymphatic outflow. The pelvic phase (Figure 5) is executed using four plus one service ports. An 8 mm periumbilical port is placed on the contralateral side of the treatment area. Following the creation of the pneumoperitoneum at a pressure of 8 mmHg, three supplementary 8 mm ports are inserted under laparoscopic visualization along a line connecting the contralateral anterosuperior iliac spine to the region of the contralateral hypochondrium. A 12 mm service port is then positioned on the ipsilateral side. During the robotic docking phase, the optic instrument is directed towards the inner inguinal ring. Following the attachment of robotic arms to the trocars, instruments are inserted. Monopolar forceps are employed for dissection.

**Figure 6:**
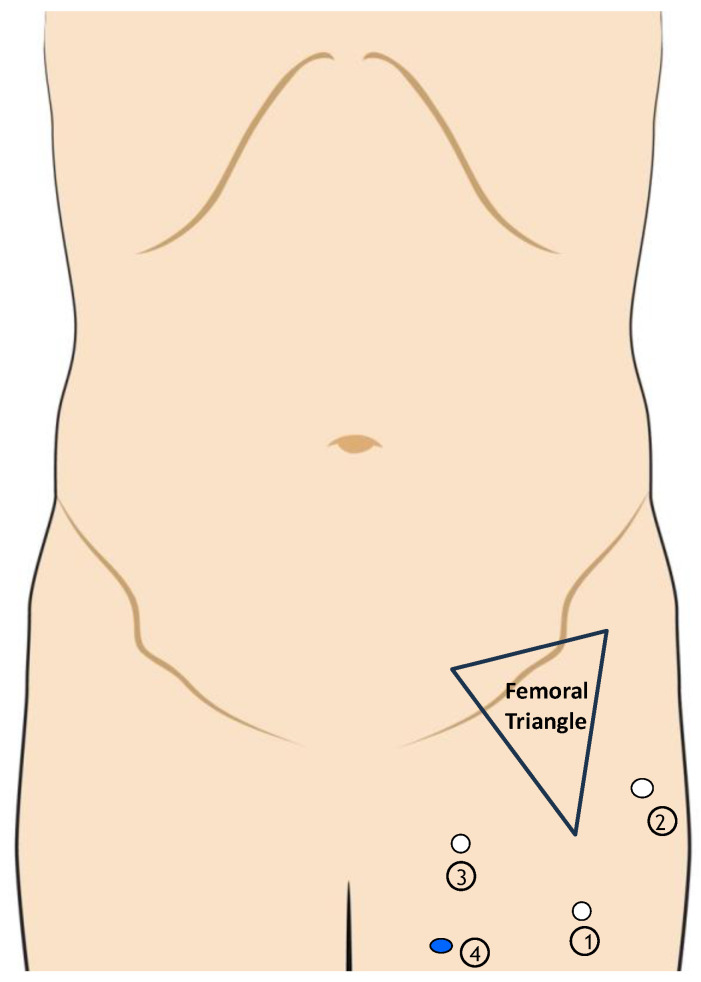
Port placement during the inguinal step of the robotic procedure. 1: 10 mm port, 3 cm above the apex of femoral triangle. 2–3: 8 mm port, 3 cm above the medial and the lateral boundaries of the femoral triangle. 4: 12 mm service port.

Regarding the inguinal phase (Figure 6), three primary service ports and one auxiliary port are utilized. A 10 mm port is positioned 3 cm distal to the apex of the femoral triangle. Following the incision and dissection of Camper and Scarpa’s fascia, two further 8 mm robotic ports are placed 3 cm beyond the medial and lateral borders of the femoral triangle, while a 12 mm port is situated beneath the lateral port. A new docking step is executed.

## 2. Results

This work presents a literature review of six papers [4,5,6,7,8,9,10,11] that detail the authors’ experiences with RIIOL (*n* = 64) and six studies [12,13,14,15,16,17] discussing the experience with videoscopic procedures (*n* = 187) in patients with cutaneous melanoma. The perioperative outcomes evaluated included the median lymph node yield, median positive lymph nodes, the surgical duration (measured in minutes), the median hospital stay, and complication rates post-procedure, as indicated by the Clavien–Dindo Score (Table 1 and Table 2). The outcomes were also assessed for the evaluation of the open technique (*n* = 83) [6,7,8,15] (Table 3).

Comparing 22rPLNDs and 41 oPLNDs, Miura et al. reported similar lymph node retrieval, basin recurrence, and complication rates, and a shorter length of stay (LOS)—2 vs. 4 days in the robotic group, thus saving 5625$ per patient. Dossett et al. compared 13 rPLNDs and 25 oPLNDs that were treated for 5 years. Similarly, they reported equivalent lymph node retrieval and complication rates and operative times, and a shorter LOS (2 vs. 4 days), with average costs of 9867$ vs. 15,492$. It is unclear, however, why the shorter stay provides equal complication rates in both studies (possible bias). Most recently, Roshan et al. (8 oPLNDs and 14 rPLNDs, study period 2012–2023) reported a similar outcome. The oncological outcomes (Table 4) were measured by basin recurrence and distant spread, recurrence-free survival (RFS), and overall survival (OS). Outcomes were similar in both procedures [6,7,8]. The comparison of robotic and videoscopic dissection yields almost the same results (Table 1 and Table 3). 

Summarizing the median values of the reported variables, we found comparable characteristics, except for the shorter LOS in rPLND (Table 5).

## 3. Discussion

After the introduction of the first endoscope prototypes and the first laparoscopic appendectomy in 1980, minimally invasive surgery has become a standard of care. In this revolutionary process, the robotic approach represents the next step: it allows us to address most of the technical limitations of conventional laparoscopy with better visualization and precision. Since the first robotic system (PUMA 560) was used for a brain biopsy in 1985, intense technological improvement has been observed and the new emerging platforms, such as DaVinci X, DaVinci Xi, and the Hugo RAS System, allow for a significantly different method of operating. 

Melanoma is a neoplasm whose incidence has been increasing in the last decades, especially in the Western world.

NCCN 2019 suggests the use of IIOL in the case of clinically positive superficial nodes or >3 positive superficial nodes, if pelvic CT scan is positive or if Cloquet’s lymph node is positive.

In a study of 134 patients followed up for 39 months, Eggers et al. demonstrated that “the addition of an iliac/obturator dissection to an inguinal dissection for both microscopic and macroscopic nodal disease did not significantly affect lymph node recurrence rates, disease-free survival, or overall survival” [18].

In a large study, Verver et al. confirmed that “There was no significant difference in recurrence pattern and survival rates between patients undergoing inguinal or ilioinguinal dissection after a positive SNB, even after stratification for a positive completion LND result” [19].

However, the recent guidelines for melanoma modified the indications for IIOL. According to AIOM guidelines [1] on cutaneous melanoma, complete lymph node dissection is the treatment of choice in patients with clinically and/or radiologically involved lymph nodes. 

According to the National Comprehensive Cancer Network (NCCN) practice guidelines 2024 pelvic lymph node dissection is also indicated in clinically positive LNs after neoadjuvant immunotherapy, targeted therapy, or both [2].

IIOL plays an important role in the management of patients with melanoma, penile, and vulvar cancer, but it is associated with possible intraoperative and postoperative complications. IIOL dissection has resulted in higher morbidity than axillary and cervical LN dissection; in fact, the prevalence of lymphedema after axillary LN dissection is 10%, while it is up to 35% after IIOL [20].

Intraoperative complications may include arterial and venous damage, as well as femoral nerve injury. Potential postoperative consequences include wound dehiscence, cutaneous infection (superficial and/or deep), incisional skin edge necrosis, skin flap necrosis, seroma, lymphoma, and chronic lymphedema. The incidence of severe complications ranges from 50% to 90% in cases of open dissection, significantly affecting patients’ quality of life [3]. 

The high rate of wound complications after IIOL can be explained by the postoperative collection of fluid in the wound due to the damage done to lymphatic vessels. Different patient- and surgery-related risk factors have been proposed, such as diabetes, smoking, obesity, and a long operative time [21]. These risk factors might influence the choice of surgical procedure, but they are not considered in the selection of the surgery procedure proposed in the studies included in this review.

In the absence of guidelines on the therapeutic approach to lymphatic complications after IIOL, various strategies have been suggested to mitigate these complications: possible interventions include the instillation of fibrin glue [22], talcum [23], doxycycline [24], erythromycin [25], and a low dose of radiotherapy [26]. These interventions have the aim of assessing the obliteration of the leaking lymphatic channel. But, in the era of minimally invasive technologies, video laparoscopic (VIL) and robotic-assisted (RIIOL) techniques are used in IIOL procedures.

The significance of robotic approaches for intraoperative imaging in melanoma patients remains contentious; multiple studies have revealed preliminary results for both video laparoscopic and robotic-assisted operations.

The quality of the available literature is very low. Only three of the included studies historically compared robotic and open PLND [6,7,8]. In the study of Miura et al., the indications for PLND were the involvement of >3 inguinal LNs, inguinal metastasis >3 cm, and the identification (but not sampling) of pelvic sentinel LNs via lymphoscintigraphy in the setting of positive superficial LNs of metastatic pelvic disease. Open PLND was utilized in cases with bulky regional disease and a history of extensive intra-abdominal operations. The comparison may be compromised because the study encompasses a large period (2006–2018). 

The complication rate post-procedure is indicated by the Clavien–Dindo Score, a score used to classify the postoperative complications based on the procedures adopted to correct these complications. Our review showed that the complication rates classified as Clavien–Dindo 1 according to the Clavien–Dindo Score are similar for the minimally invasive (videoscopic and robotic) procedure and open procedure. Instead, the complication rate classified as Clavien–Dindo 2 is higher for robotic procedure compared to open and videoscopic IIOL. However, the robotic procedure presents a lower rate of complications classified as Clavien–Dindo 3 compared both to open and videoscopic IIOL (Table 5).

A brief summary of the pros and cons of the various surgical procedures that emerge from our study is shown in Table 6.

The limitations of the present study are the retrospective cohort design of the included studies, and the limited number of patients treated with the robotic-assisted approach compared to the open dissection. The long study period may result in a bias linked to the evolving systematic therapy. These constraints may result in the challenges linked to the robotic process being underappreciated, thus necessitating future investigations to validate these first findings. 

## 4. Conclusions

Nowadays, PLND is only indicated in radiologically detected metastases. rPLND is feasible and carries similar complication rates and oncological outcomes. The reason for the shorter LOS is not clear but is associated with lower hospital costs. Unfortunately, the quality of evidence is too low to draw any valid conclusions. It is probable that, from a surgeon’s point of view, the robotic techniques offer several advantages over videoendoscopic techniques such as three-dimensional imaging, ergonomic control, and tools that mimic human hand movements. Randomized controlled trials are necessary to validate the benefits of RIOL compared to VIL and IIOL, but the recruitment rate is very low because of the restricted indications for PLND against the background of the continuously evolving system therapy.

## Figures and Tables

**Table 1:** Studies present in the literature and the outcomes of the robotic procedure.

Author	N of Patients	Histology	Lymph Node Yield, Median	Positive Lymph Nodes Median	Hospital Days	Surgery Time (Min; Median)	Complications by Clavien–Dindo Score 1	Complications by Clavien–Dindo Score 2	Complications by Clavien–Dindo Score > 3
Miura [7]	22	Melanoma	11 (8.2–12)	1 (0.1)	N/A	199 (168–229.2)	40.9%	4.6%	0%
Roshan [6]	14	Melanoma	6.0 (3.75–9.0)	2 (1.0–3.0)	1.98 (1.39–3.50)	126 (97.8–161)	35.7%	50%	7.1%
Hyde [9]	4	Melanoma	5.5 (1–14)	N/A	2 (1–3)	192 (120–231)	0%	50%	0%
Sanchez [11]	1	Melanoma	8	0	2	130	0%	0%	0%
Francone [4]	10	Melanoma (8) and MMC (2)	15.2 (5–27)	N/A	2	330	30%	10%	N/A
Dossett [8]	13	Melanoma	11 (5–16)	0 (0–5)	2 (1–3)	227	31%	0%	0%

**Table 2:** Studies present in the literature and the outcomes of the videoscopic procedure.

Author	N of Patients	Histology	Lymph Node Yeld, Median	Positive Lymph Nodes, Median	Hospital Days	Surgery Time (Min, Median)	Conversion to Open Procedure	Complications by Clavien–Dindo Score 1	Complications by Clavien–Dindo Score 2	Complications by Clavien–Dindo Score > 3
Sommariva [12]	48	Melanoma	N/A	40 (80%)	6 (5–7)	255 (231–278)	6 (11%)	15 (30%)	4 (8%)	3 (6%)
Solari [15]	24	Melanoma	24.1 (8–38)	1.58 (6.58%)	2 (1–3)	302	N/A	16.5%	N/A	N/A
Sommariva [14]	24	Melanoma	21 (15–25)	1 (1–2)	7 (5–8)	270 (245–300)	4	11	5	1
Vrielink [13]	20	Melanoma	9.0 (1–19)	N/A	4.0 (2.0–5.0)	110 (79.0–165-0)	N/A	5 (31.3%)	6 (37.7)	5 (31.3)
Khan [17]	26	Melanoma; MCC; penile cancer	9.8 +/− 3.7	1 (0–9)	N/A	119 (89–160)	0	4	7	6
Delman [16]	45	Melanoma; MCC; penile cancer	11 (4–24)	N/A	3.1	165 (75–245)	2	8	N/A	N/A

**Table 3:** Studies present in the literature and the outcomes of the open procedure.

Author	N of Patients	Histology	Lymph Node Yeld, Median	Positive Lymph Nodes, Median	Hospital Days	Surgery Time (Min, Median)	Complications by Clavien–Dindo 1	Complications by Clavien–Dindo 2	Complications by Clavien–Dindo > 3
Miura [7]	30	Melanoma	9 (8–13)	2 (0–4)	4	214.5 (151.5–250.2)	10 (24.4)		0
Roshan [6]	8	Melanoma	6.5 (6.0–12.5)	2.5 (1.25–3.75)	5.34 (3.77–6.94)	174 (158–216)	1 (12.5)	4 (50)	2 (25)
Dossett [8]	25	Melanoma	10 (5–16)	1 (0–8)	4 (1–7)	230	11 (44)	1 (4)	1 (4)
Solari [15]	20	Melanoma	15.5 (11–28)	3.15 (15.75%)	6 (4–8)	190	6 (30%)	3 (13)	0

**Table 4:** Oncological outcomes.

Author	Median FU (Months)	RFS (Months)	OS (Months)	Development of Distant Disease	Basin Recurrence
Miura [7]	37.2	14.4 (rPLND) vs. 9.6 (oPLND)	42.6 (rPLND) vs. 50 (oPLND)	rPLND: 40.9% vs. oPLND: 43.9%	rPLND: 4.5% vs. oPLND: 7.3%
Roshan [6]	oPLND: 25.7 vs. rPLND: 21.1	N/A	N/A	rPLND: 71.4% vs. oPLND: 75.0%	rPLND: 42.9% vs. oPLND: 37.5%
Dossett [8]	oPLND: 25.7 vs. rPLND: 21	N/A	N/A	rPLND: 23% vs. oPLND: 44%	rPLND: 0% vs. oPLND: 4%
Sommariva [12]	28.0	9 (4–17.8)	N/A	VIL: 27.5 %	VIL: 23.5%
Sommariva [14]	18.0	N/A	N/A	VIL: 13%	VIL: 8.7%

**Table 5:** Comparison of the three techniques used for LND.

	LN Retrieval, *n*	LOS (Days)	Surgery Time, Min	Clavien–Dindo 1,%	Clavien–Dindo 2, %	Clavien–Dindo 3, %
Robotic	5–15	2	126–330	31–41	5–50	7
Videoscopic	9–24	3–7	165–270	17–31	8–38	6–31
Open	6–15	4–6	174–215	13–44	4–50	4–25

**Table 6:** Cons and pros of robotic, videoscopic, and open IIOL.

Robotic	Videoscopic	Open
Lower LOS	Lower LOS than open	Higher LOS
Lower C-D3 complication rates	Lower C-D 2 complication rates than robotic	Higher C-D 3 complication rates
Lower hospital cost	Lower surgical time than robotic	Higher hospital cost
Higher C-D 1 complication rates	Higher hospital cost than robotic	Lower surgical time than robotic
Higher surgical time	Higher C-D 3 complication	

## Data Availability

The data used to support the findings of this study are included within the article. Further enquiries can be directed to the corresponding author.
